# CCL2 deficient mesenchymal stem cells fail to establish long-lasting contact with T cells and no longer ameliorate lupus symptoms

**DOI:** 10.1038/srep41258

**Published:** 2017-01-24

**Authors:** Hong Kyung Lee, Hyung Sook Kim, Ji Sung Kim, Yong Guk Kim, Ki Hwan Park, Jae Hee Lee, Ki Hun Kim, In Young Chang, Sang-Cheol Bae, Youngsoo Kim, Jin Tae Hong, John H. Kehrl, Sang-Bae Han

**Affiliations:** 1 College of Pharmacy, Chungbuk National University, Cheongju, Chungbuk 28160, Republic of Korea; 2 Corestem Inc, Gyeonggi 13486, Republic of Korea; 3 Hanyang University Hospital for Rheumatic Diseases, Seoul 04763, Republic of Korea; 4 National Institute of Allergy and Infectious Diseases, National Institutes of Health, Bethesda, MD 20892, USA

## Abstract

Systemic lupus erythematosus (SLE) is a multi-organ autoimmune disease characterized by autoantibody production. Mesenchymal stem cells (MSCs) ameliorate SLE symptoms by targeting T cells, whereas the mechanisms of their efficacy remain incompletely understood. In this study, we show that transfer of human MSCs increased MRL.*Fas*^lpr^ mouse survival, decreased T cell infiltration in the kidneys, and reduced T cell cytokine expression. *In vitro*, allogeneic mouse MSCs inhibited MRL.*Fas*^lpr^ T cell proliferation and cytokine production. Time-lapse imaging revealed that MSCs recruited MRL*.Fas*^lpr^ T cells establishing long-lasting cellular contacts by enhancing T cell VCAM-1 expression in a CCL2-dependent manner. In contrast, CCL2 deficient MSCs did not induce T cell migration and VCAM-1 expression, resulting in insufficient cell-cell contact. Consequently, CCL2 deficient MSCs did not inhibit IFN-γ production by T cells and upon transfer no longer prolonged survival of MRL*.Fas*^lpr^ mice. Taken together, our imaging study demonstrates that CCL2 enables the prolonged MSC–T cell interactions needed for sufficient suppression of autoreactive T cells and helps to understand how MSCs ameliorate symptoms in lupus-prone MRL*.Fas*^lpr^ mice.

A hallmark of systemic lupus erythematosus (SLE) is the production of autoantibodies to ubiquitous self-antigens^1^. Inflammation and end-organ damage are driven by autoantibody immune complex deposition. In the kidney, this leads to lupus nephritis, a serious complication that occurs in more than 50% of SLE patients^2^,^3^. Standard treatment regimens for SLE patients include immunosuppressive drugs, such as cyclophosphamide and mycophenolate mofetil^4^. While some patients respond well to these medications, others do not. Furthermore, long-term usage of these drugs raises the incidence of serious infections, cardiovascular diseases, and cancer^5^,^6^. Thus, additional therapeutic options are needed for SLE patients and others who suffer from serious autoimmune disorders^4^.

In the past decade, mesenchymal stem cells (MSCs) have emerged as a promising new therapy for the treatment of SLE^7^. MSCs are adult stem cells isolated from various human tissues including bone marrow, adipose tissue, umbilical cord blood, and skeletal muscle; MSCs can differentiate into various cell types and can potentially replace damaged cells *in vivo*^4^,^8^,^9^. MSCs produce soluble factors that support angiogenesis, enhance the growth of other stem cells, and reduce apoptosis; all of these effects can facilitate tissue regeneration^10^. Another important aspect of MSC therapy relevant to SLE and other autoimmune illnesses is their effects on immune cell functions. MSCs suppress T cell proliferation and cytokine production, reduce B cell proliferation and antibody secretion, decrease the generation and function of dendritic cells, and reduce the activity of natural killer cells^4^,^10^,^11^,^12^,^13^,^14^. MSCs also enhance the activity of regulatory T (Treg) cells^15^. MSCs infiltrate damaged tissues and, since they express low MHC-I levels and lack MHC-II, they can escape immune recognition and clearance^16^. MSCs are thought to inhibit T cell functions by two different mechanisms: by producing soluble mediators and by direct cell–cell contacts. The soluble immunosuppressive factors produced by MSCs include IL-10, nitric oxide (NO), tumor growth factor (TGF)-β, prostaglandin E_2_ (PGE_2_), and indoleamine 2,3-dioxygenase (IDO), all of which can inhibit the functions of major immune cells^10^,^11^,^12^,^17^,^18^,^19^. Overall, the unique properties of MSCs make them good candidates for the treatment of SLE and other serious autoimmune diseases^20^.

MRL/MpJ-*Fas*^*lpr*^ (called MRL.*Fas*^lpr^ hereafter) mice lack *Fas* and spontaneously develop an SLE-like disease^21^. The onset and symptom severity in these mice depend on their genetic background. Female MRL*.Fas*^lpr^ mice die at an average age of 17 weeks and males at 22 weeks. Similar to SLE patients, MRL*.Fas*^lpr^ mice have a marked increase in anti-dsDNA antibodies in their blood and develop severe nephritis. Zhou *et al*. examined the impact of a single injection of 1 × 10^6^ human bone marrow–derived MSCs into 16-week-old female MRL*.Fas*^lpr^ mice. Adoptively transferred MSCs reduced mortality and decreased the levels of anti-dsDNA antibodies and the extent of renal pathology at 32 weeks^21^. Ma *et al*. reported that adoptive transfer of 1 × 10^6^ allogenic MSCs (Balb/c) to 18-week-old MRL*.Fas*^lpr^ mice increased their survival at week 26 and ameliorated nephritis by inhibiting B cell activation. The mice that received MSCs had lower levels of B cell activating factor (BAFF) in their kidneys and spleens^22^. Several pilot studies in China have indicated that human allogenic MSCs can induce clinical remission and improve organ dysfunction in drug-resistant SLE patients^23^.

MSCs from a variety of tissue sources are being evaluated in over 200 clinical trials for the treatment of a variety of illnesses including SLE^24^. Yet, much remains to be learned about how these cells home, engraft, and function. In this study, we focused on the mechanisms by which MSCs limit T cell functions at a single-cell level. To help explain how MSCs improve immune dysfunction in SLE, we examined the contact dynamics between MSCs and T cells, including T cell migration and motility, and contact frequency, duration, and timing. We found that MSCs attract activated T cells and form long-lasting contacts with them, and that these contacts are necessary for the suppressive activity of MSCs.

## Results

### MSCs ameliorate SLE development in MRL.*Fas* ^lpr^ mice

**Figure 1:**
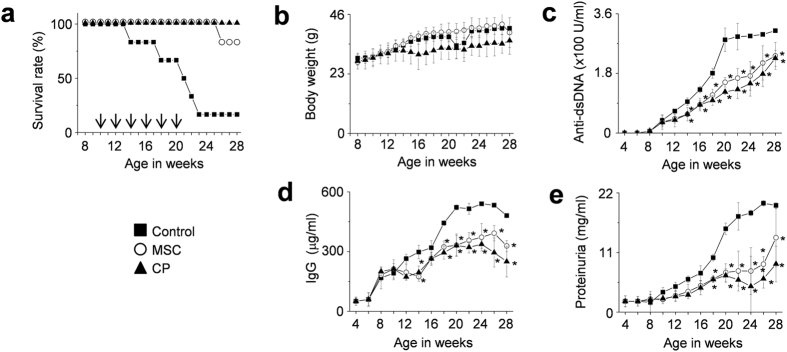
MSCs ameliorate SLE development in MRL.*Fas*^lpr^ mice (multiple injections). MRL*.Fas*^*lpr*^ mice were intravenously injected with vehicle (control, n = 5), MSCs (1 × 10^6^ cells/injection, n = 6), or cyclophosphamide (CP, 50 mg/kg, n = 6) 6 times (arrows) every two weeks from the age of 10 weeks. (**a,b**) Survival (**a**) and body weight (**b**) were measured every week. (**c**–**e**) Serum and urine were collected every two weeks. The levels of anti-dsDNA IgG (**c**) and total IgG (**d**) in serum and the level of proteinuria (**e**) were measured. *p < 0.01 versus control.

We first verified the therapeutic activity of human MSCs in the MRL.*Fas*^lpr^ mouse model by using a more aggressive adoptive transfer protocol than previously employed^22^,^25^,^26^. We generated MSCs from human bone marrow cells and injected them intravenously into female MRL*.Fas*^lpr^ mice (1 × 10^6^ cells/injection). The mice received a total of six cell transfers every two weeks from 10 weeks of age. Another group of mice received cyclophosphamide (CP, 50 mg/kg) every two weeks. MSCs significantly prolonged survival: 90% of the mice receiving MSCs survived up to 28 weeks of age, whereas only 10% of the control mice survived (Fig. 1a). MSCs did not affect body weight (Fig. 1b), and no untoward effects were noted. The serum level of anti-dsDNA (Fig. 1c) and total IgG antibodies (Fig. 1d), and the amount of protein in the urine (Fig. 1e) all decreased in MSC-treated mice when compared with control mice. Although CP also ameliorated the disease, it slightly interfered with the normal increase in body weight, which was however not significantly different from that of control mice. Despite the aggressive cell transfer protocol, the suppression of disease in MRL*.Fas*^lpr^ mice did not differ substantially from that reported in previous studies^22^,^25^,^26^.

**Figure 2:**
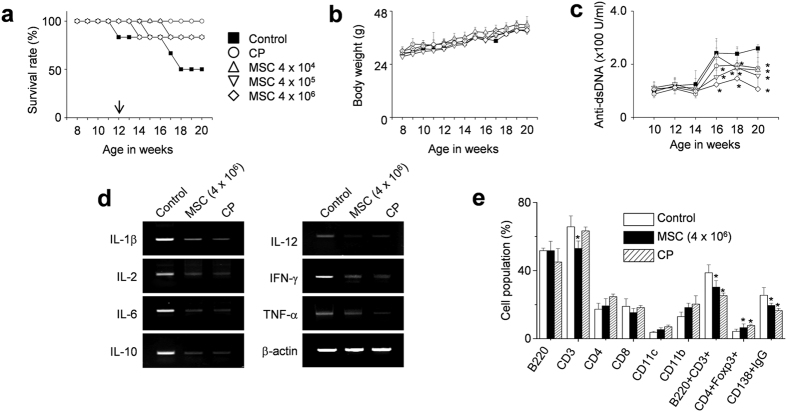
MSCs ameliorate SLE development in MRL.*Fas*^lpr^ mice (single injection). MRL*.Fas*^lpr^ mice were intravenously injected with vehicle (control, n = 5), MSCs (4 × 10^4^ cells, 4 × 10^5^ cells, or 4 × 10^6^ cells/injection, n = 5), or cyclophosphamide (CP, 50 mg/kg, n = 5) once at the age of 12 weeks. (**a**–**c**) Survival (**a**) and body weight (**b**) were measured every week. The serum levels of anti-dsDNA IgG were measured every two weeks (**c**). (**d,e**) Mice were sacrificed at the age of 20 weeks and spleens were isolated. Total RNA was isolated from spleen cells and the expression of inflammatory cytokine genes was examined by RT-PCR (**d**). Subset ratios were analyzed by flow cytometry (**e**). *p < 0.01 versus control.

Therefore, we reverted to a single adoptive transfer of human MSCs (4 × 10^4^, 4 × 10^5^, or 4 × 10^6^ cells/injection) into 12-week-old female MRL*.Fas*^lpr^ mice. MSCs at all doses prolonged survival without body weight loss (Fig. 2a and b). The serum level of anti-dsDNA antibodies also decreased in MSC-treated mice when compared with control mice (Fig. 2c). At 20 weeks of age, when 50% of control mice had survived, we isolated spleen cells from control mice and mice treated with 4 × 10^6^ MSCs. The expression of all inflammatory cytokines tested (IL-1β, IL-2, IL-6, IL-10, IL-12, IFN-γ, and TNF-α) decreased in the spleens of MSC-treated mice when compared with those from control mice (Fig. 2d). The frequency of CD4^+^Foxp3^+^ Treg cells increased and that of CD138^+^IgG^+^ plasma cells decreased in the spleens of MSC-treated mice (Fig. 2e). The infiltration of neutrophils, macrophages, dendritic cells, B cells, and T cells into the kidney was examined histopathologically (Supplementary Fig. S1). In control mice, these cells were not detected in the kidney before disease onset (5 weeks of age); their infiltration strongly increased with disease progression (20 weeks of age) and was dose-dependently decreased in MSC-injected mice (Supplementary Fig. S1). The infiltration of Treg cells decreased with the progression of disease in control mice, and this trend was reversed by MSC transfer (Supplementary Fig. S1). These data show that the transfer of human MSCs ameliorates the development of SLE-like disease in MRL*.Fas*^lpr^ mice. Furthermore, a single adoptive transfer of human MSCs appears to be enough to reduce end-organ damage and decrease mouse mortality.

### MSCs show soluble factor- and contact-dependent inhibition of T cells

**Figure 3:**
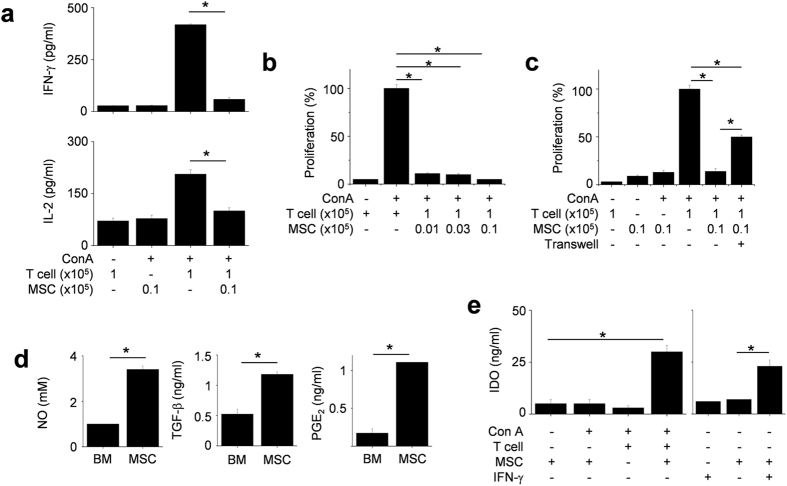
MSCs inhibit T cells via soluble factor- and contact-dependent mechanisms. (**a,b**) T cells (1 × 10^5^ cells/well) were activated by the T cell-specific mitogen concanavalin A (Con A, 1 μg/ml) in the presence or absence of MSCs (0.1 × 10^5^ cells/well) for 72 h (n = 3). The levels of IFN-γ and IL-2 in culture medium were measured by ELISA (**a**) and T cell proliferation was measured by the mitogen assay (**b**). *p < 0.01. (**c**) MSCs (0.1 × 10^5^ cells/well) were added to the upper wells and T cells (1 × 10^5^ cells/well) to the lower wells to avoid cell–cell contact in transwell plates having a 5 μm insert. MSCs (0.1 × 10^5^ cells/well) and T cells (1 × 10^5^ cells/well) were also added to the lower wells to ensure cell–cell contact. After incubation with Con A for 72 h, the mitogen assay was performed (n = 3). *p < 0.01. (**d**) The levels of NO, TGF-β, and PGE_2_ accumulated in culture medium of MSCs for 24 h were measured. *p < 0.01 (n = 3). (**e**) MSCs (0.1 × 10^5^ cells/well) and T cells (1 × 10^5^ cells/well) were co-cultured in the presence of Con A for 24 h. Another experiment, MSCs were treated with only IFN-γ (100 U/ml) for 24 h. The levels of IDO were measured with ELISA (n = 3). *p < 0.01.

Next, we investigated the mechanisms of MSC-based immunotherapy with a major focus on the inhibition of T cell functions. *In vitro* mechanism studies, we used allogeneic MSCs isolated from bone marrow cells of Balb/c mice and splenic T cells isolated from MRL*.Fas*^lpr^ mice. Balb/c MSCs reduced nephritis in MRL*.Fas*^lpr^ mice, as previously noted^22^. We found that allogenic murine MSCs inhibited IFN-γ and IL-2 production by MRL*.Fas*^lpr^ T cells (Fig. 3a) and reduced T cell proliferation (Fig. 3b). To assess the role of soluble factors in these effects, we used a transwell assay. We added Balb/c MSCs to the upper wells and MRL*.Fas*^lpr^ T cells to the lower wells, thereby avoiding direct cell–cell contact. MSCs inhibited T cell proliferation, although less efficiently than in co-culture experiments, implying soluble factor-dependent inhibition mechanism (Fig. 3c). We detected the presence of NO, TGF-β, and PGE_2_ in the cell supernatants (Fig. 3d). Although naïve MSCs did not express IDO, they produced IDO following co-culture with concanavalin A (Con A)-activated T cells or murine IFN-γ (Fig. 3e). These data indicate that MSCs inhibit T cell functions via direct contact as well as by producing soluble factors.

### MSCs produce CCL2 to induce T cell migration

**Figure 4:**
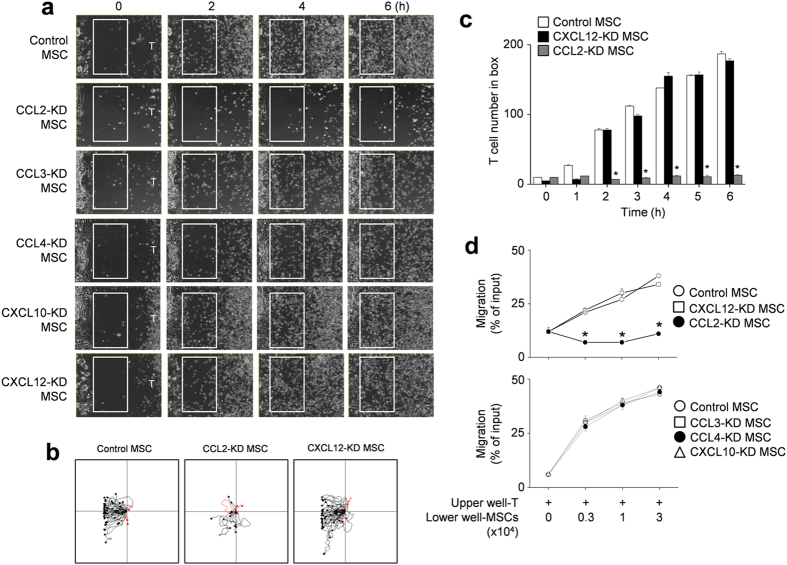
T cell migration towards CCL2-KD MSCs. (**a**–**c**) Balb/c MSCs were transfected with negative (control), CCL2, CCL3, CCL4, CXCL10, or CXCL12 siRNA. T cells were purified from spleen cells of MRL*.Fas*^lpr^ mice. For time-lapse imaging, MSCs (70 μl of 0.3 × 10^6^ cells/ml) were seeded into the left chamber and T cells (70 μl of 3 × 10^6^ cells/ml) into the right chamber of culture-insert μ-Dish^35mm^ culture dishes. Images were acquired every 2 min for 6 h (n = 3). Representative snapshots (**a**), the tracks of T cells in white boxes (**b**), and the number of T cells passing through the white boxes are shown (**c**). *p < 0.01 versus control. (**d**) Control or chemokine-knockdown (KD) MSCs (0.3 to 3 × 10^4^ cells/well) were added to the lower wells and MRL*.Fas*^lpr^ T cells (1 × 10^4^ cells/well) to the upper wells of transwell plates having a 5 μm insert. After 1.5 h, the number of T cells migrating to the lower well was determined (n = 3). *p < 0.01 versus control.

**Figure 5:**
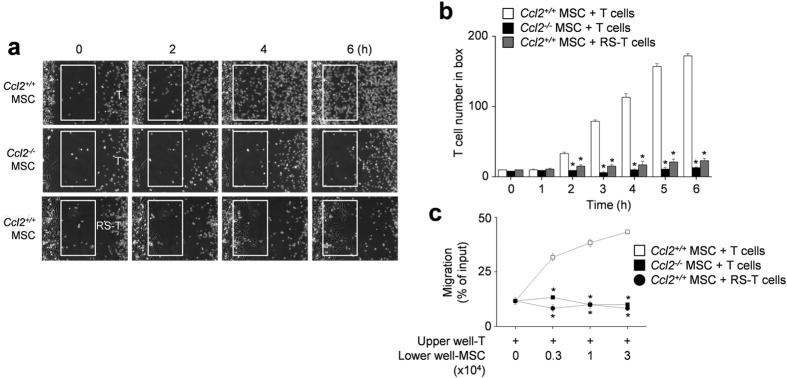
T cell migration towards *Ccl2*^*-*/*-*^ MSCs. (**a**,**b**) For time-lapse imaging, MSCs (70 μl of 0.3 × 10^6^ cells/ml) were seeded into the left chamber and T cells (70 μl of 3 × 10^6^ cells/ml) into the right chamber of culture-insert μ-Dish^35mm^ culture dishes. MSCs were generated from bone marrow cells of C57BL/6 (*Ccl2*^+/+^) and *Ccl2*^−/−^ mice and T cells were purified from spleen cells of MRL*.Fas*^lpr^ mice. T cells were also pretreated with 30 μg/ml RS102895 for 1 h. Images were acquired every 2 min for 6 h (n = 3). Representative snapshots (**a**) and the number of T cells passing through the white boxes (**b**) are shown. *p < 0.01 versus control. (**c**) *Ccl2*^+/+^ or *Ccl2*^−/−^ MSCs (0.3 to 3 × 10^4^ cells/well) were added to the lower wells and MRL*.Fas*^lpr^ T cells (3 × 10^4^ cells/ml) to the upper wells of transwell plates having a 5 μm insert. T cells were also pretreated with RS102895 (RS, 30 μg/ml) for 1 h. After 1.5 h, the number of T cells migrating to the lower well were determined (n = 3). *p < 0.01 versus *Ccl2*^+/+^ MSC and T cell group.

Next, we focused on how MSCs contacted T cells. To determine whether allogenic MSCs actively attracted T cells, we first assessed the expression profiles of chemokines and chemokine receptors that are likely to mediate T cell migration^27^,^28^. We found that MSCs expressed mRNAs for CCL2, CCL3, CCL4, CXCL10, and CXCL12, and CCL2 and CXCL12 proteins were detectable by ELISA in conditioned supernatants (Supplementary Fig. S2). MRL*.Fas*^lpr^ T cells expressed mRNAs that encoded the chemokine receptors CCR2, CCR5, and CXCR4, and we detected the respective proteins in cell lysates by immunoblotting (Supplementary Fig. S2). Next, we used small interfering RNAs (siRNAs) to knockdown (KD) chemokine expression in MSCs (Supplementary Fig. S2) and performed time-lapse imaging to assess T cell migration towards control and KD MSCs. We placed MSCs on the left side of an imaging chamber and MRL.*Fas*^lpr^ T cells on the right side and acquired images every 2 min for 6 h. Representative images collected at 2-h intervals are shown in Fig. 4a. While control, CCL3-, CCL4-, CXCL10-, and CXCL12-KD MSCs induced T cell migration towards MSCs, CCL2-KD MSCs did not induce T cell migration (Supplementary movies S1 and S2 and Fig. 4a). Tracks of T cells in the outlined regions showed the leftward migration of T cells (Fig. 4b). These results were quantitated at each time point (Fig. 4c). We confirmed these results using transwell assays, which showed that CCL2-KD MSCs did not induce the migration of MRL*.Fas*^lpr^ T cells (Fig. 4d). In addition, *Ccl2*^−/−^ (B6.129S4-*Ccl2*^*tm1Rol*^/J C57BL/6) MSCs failed to attract T cells (Supplementary movie S3 and Fig. 5a) and T cells treated with the CCR2 antagonist RS102895 showed little migration towards control MSCs (Supplementary movie S4 and Fig. 5a). These results were quantitated at each time point (Fig. 5b) and transwell assays showed that inhibition of the CCL2–CCR2 axis blocked the migration of MRL*.Fas*^lpr^ T cells towards MSCs (Fig. 5c). We also checked that the highest concentration of RS102895 did not affect T cell viability (data not shown). These data show that murine MSCs produce CCL2, which induces migration of CCR2-expressing MRL*.Fas*^lpr^ T cells near MSCs.

Next, we examined whether human MSCs recruit T cells by producing CCL2. We placed human MSCs on the left side of an imaging chamber and MRL.*Fas*^lpr^ T cells on the right side and acquired images every 2 min for 6 h. Representative images collected at 2-h intervals are shown in Supplementary Fig. S3a. While control MSCs induced T cell migration, CCL2-KD MSCs did not (Supplementary movies S5 and S6 and Fig. S3a). The number of T cells passing through the white box at each time point is shown in Supplementary Fig. S3b. Overall, these data show that murine and human MSCs produce CCL2 to recruit CCR2-expressing MRL*.Fas*^lpr^ T cells.

### CCL2 increases the duration of the MSC–T cell contact

**Figure 6:**
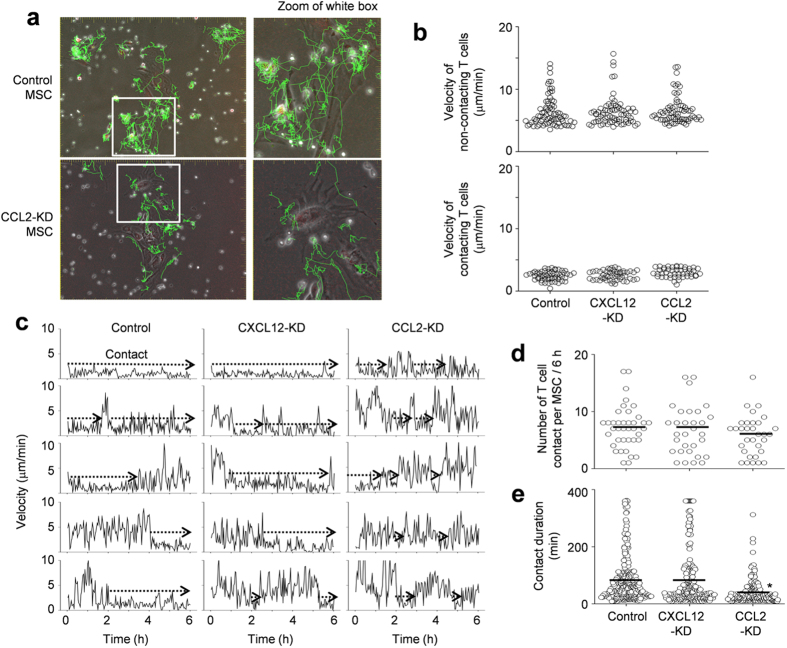
Contact dynamics between MSCs and T cells. Control, CXCL12-KD, and CCL2-KD MSCs were labeled with CMTMR (red). MRL*.Fas*^lpr^ T cells were labeled with CFSE (green). MSCs (0.1 × 10^5^ cells/well) and T cells (1 × 10^5^ cells/well) were mixed and added onto 35-mm culture dishes. Dishes were preincubated for 1 h under the microscope and images were acquired in three channels (phase contrast; CFSE, green filter; and CMTMR, red filter) every 2 min for 6 h (six movies from three independent experiments per group). (**a**) Representative images (original magnification, 100x; zoomed, 2.3×). (**b**) Instantaneous velocities of non-contacting T cells (n = 78, 76, and 74 from the left) and contacting T cells (n = 58, 53, and 48 from the left). (**c**) Representative profiles of T cell velocity. (**d**) The number of T cell contacts per MSC (n = 39, 31, and 32 from the left). (**e**) Contact duration between T cells and MSCs (n = 281, 240, and 190 from the left). *p < 0.01 versus control. Bars represent the mean of the data.

To test whether CCL2 produced by MSCs affected MSC–T cell interactions, we placed MSCs and MRL*.Fas*^lpr^ T cells in close proximity, bypassing the need for directed T cell migration. We then imaged the contacts between T cells and control (Supplementary movies S7 and S8), CXCL12-KD, or CCL2-KD MSCs (Supplementary movies S9 and S10) every 2 min for 6 h. Representative images with superimposed tracks is shown in Fig. 6a. We found that some T cells remained in close contact with MSCs, while others moved freely with long tracks and showed little attraction to MSCs. MSCs were sessile with an average velocity of less than 1 μm/min. The non-contacting T cells had average velocities of 6–7 μm/min and contacting T cells showed oscillation movement on the surface of MSCs at average velocities of 2–3 μm/min (Fig. 6b). Average velocities of T cells were similar when they were cultured with control, CXCL12-KD, or CCL2-KD MSCs, suggesting that MSC-derived chemokines had little impact on basal T cell velocity. We also examined whether CCL2 or CXCL12 altered the likelihood of MSC contacts by assessing changes in T cell velocity (i.e., when a T cell slowed down). T cells contacted control, CXCL12-KD, and CCL2-KD MSCs at equal frequencies during the early, middle, and late stages of imaging (Fig. 6c). This suggested that the initial contacts occurred randomly and were unaffected by MSC chemokine production. T cells contacted control and CXCL12-KD MSCs on average 7 times (Fig. 6d) and each contact lasted for 79–82 min in our 6 h-imaging (Fig. 6e). T cells contacted CCL2-KD MSCs on average 6 times (similar with the number of contacts with control MSCs); however, each contact lasted only an average of 39 min in 6 h-imaging (significantly shorter than contact duration with control MSCs). T cells contacted CCL3-, CCL4-, and CXCL10-KD MSCs 8 times with a contact duration of 75–87 min, similar to control MSCs (data not shown). These data indicate that, when MSCs and T cells are in close proximity, contact frequency does not depend on CCL2, but contact duration does.

We then imaged the contacts between T cells and human MSCs (Supplementary movies S11 and S12) every 2 min for 3 h. Representative images with superimposed tracks are shown in Supplementary Fig. S4a. In comparison with control MSCs, CCL2-KD MSCs contacted T cells with a similar frequency, but showed shorter contact duration (Supplementary Fig. S4b and c).

**Figure 7:**
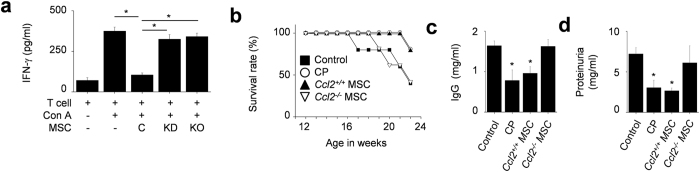
CCL2 deficient MSCs did not ameliorate SLE development in MRL.*Fas*^lpr^ mice. (**a**) MRL*.Fas*^lpr^ T cells were activated with concanavalin A (Con A) and were co-cultured with control, CCL2-KD, or *Ccl2*^−/−^ MSCs for 72 h, and the levels of IFN-γ in culture medium were measured by ELISA (n = 3). *p < 0.01. (**b–d**) MRL*.Fas*^lpr^ mice were intravenously injected with vehicle (control, n = 5), *Ccl2*^+/+^ MSCs (1 × 10^6^ cells/injection, n = 5), *Ccl2*^−/−^ MSCs (1 × 10^6^ cells/injection, n = 5), or cyclophosphamide (CP, 10 mg/kg, n = 5) once at the age of 12 weeks. Survival (**b**) was measured every week and total IgG level in serum (**c**) and protein level in urine (**d**) was measured at the age of 22 weeks. *p < 0.01 versus control.

Likely due to a shorter contact period, CCL2-KD and *Ccl2*^−/−^ MSCs did not inhibit IFN-γ production by T cells as well as control MSCs did (Fig. 7a). We also showed that the *Ccl2*^−/−^ MSCs did not prolong the survival (Fig. 7b) and did not reduce the serum IgG level (Fig. 7c) and proteinuria level (Fig. 7d) in MRL.*Fas*^lpr^ mice, although the *Ccl2*^+/+^ MSCs did. Together, these data indicate that CCL2 production by MSCs promotes the migration of MRL.*Fas*^lpr^ T cells towards MSCs and increases the duration of MSC–T cell contacts, which is apparently required for optimal inhibition of T cell functions and limiting SLE development in MRL.*Fas*^lpr^ mice.

### MSC-derived CCL2 increases VCAM-1 expression in T cells

**Figure 8:**
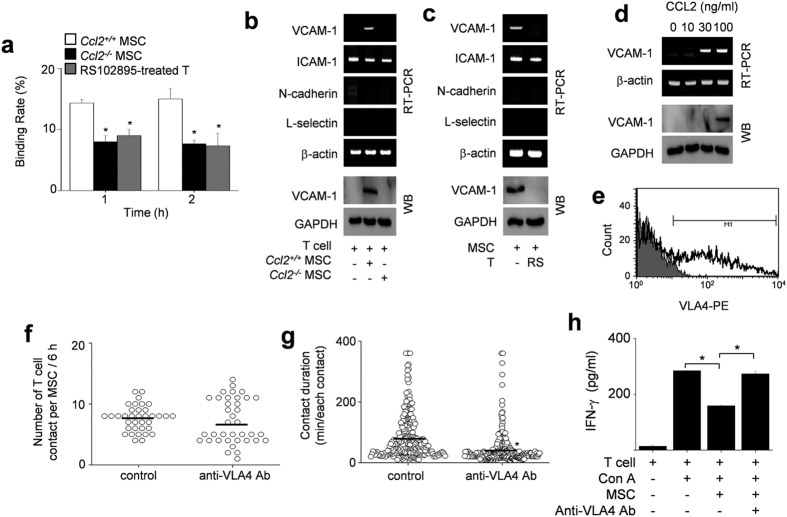
Binding rates of MSCs and T cells. (**a**) Binding rates of CMTMR-labeled MSCs (0.1 × 10^5^ cells/tube) and CMFDA-labeled T cells (1 × 10^5^ cells/tube) were analyzed by flow cytometry (n = 3). The conjugation ratio was calculated as the portion of CMFDA/CMTPX double-positive events. T cells were treated with 30 μg/ml RS102895 for 1 h. *p < 0.01. (**b,c**) MSCs (0.1 × 10^5^ cells/well) were added to the lower wells of transwell plates having a 0.4 μm insert and preincubated for 2 h. T cells (1 × 10^5^ cells/well) were then added to the upper wells and plates were incubated for 2 h (**b**). T cells were pretreated with vehicle (DMSO) or RS102895 (RS) for 1 h, washed twice and then added (1 × 10^5^ cells/well) to the lower wells (**c**). Adhesion molecule expression in T cells was assessed by RT-PCR and western blotting (n = 3). (**d**) T cells were directly treated with CCL2 for 2 h in 6-well plates, and VCAM-1 expression was assessed by RT-PCR and western blotting (n = 3). (**e**) MSCs were stained with anti-mouse VLA4 antibody conjugated with phycoerythrin and analyzed with a flow cytometer (n = 3). (**f**,**g**) MSCs were incubated with anti-mouse VLA4-neutralizing antibody for 24 h, washed twice, and mixed with T cells. Imaging was performed every 2 min for 6 h (six movies from three independent experiments). The numbers of T cell contacts per MSC (n = 36 and 37 from the left) (**f**). Contact duration between T cells and MSCs (n = 267 and 270 from the left) (**g**). *p < 0.01. (**h**) MSCs were incubated with anti-mouse VLA4 antibody for 24 h, washed twice, and mixed with T cells and 1 μg/ml Con A (added to activate T cells). After 72 h, the levels of IFN-γ accumulated in culture medium were determined by ELISA (n = 3). *p < 0.01. Bars represent the mean of the data.

To address the mechanism by which CCL2 production by MSCs increases T cell contact duration, we performed MSC–T cell binding assays. Either the use of *Ccl2*^−/−^ MSCs or interfering with CCL2 signaling by treating T cells with the CCR2 antagonist RS102895 decreased the frequency of MSC–T cell conjugate formation (Fig. 8a). As cell–cell interactions often depend on the expression of cell adhesion molecules, such as integrins, cadherins, and selectins^29^,^30^, we assessed the expression of several such molecules by RT-PCR and immunoblotting. We found that co-culture of T cells with control MSCs increased T cell expression of VCAM-1, but not ICAM-1, N-cadherin, or L-selectin, whereas co-culture with *Ccl2*^−/−^ MSCs failed to increase T cell VCAM-1 expression (Fig. 8b) and control MSCs did not increase VCAM-1 expression in T cells treated with the CCR2 antagonist RS102895 (Fig. 8c). Consistent with these data, soluble CCL2 increased VCAM-1 expression in MRL*.Fas*^*lpr*^ T cells (Fig. 8d) and MSCs clearly expressed integrin α_4_β_1_ (VLA-4, a ligand of VCAM-1) (Fig. 8e). Finally, we tested whether interfering with the interaction between VLA-4 and VCAM-1 would affect the contacts between MSCs and T cells. Addition of a VLA-4-neutralizing antibody strongly decreased contact duration (Fig. 8g) without changing contact frequency (Fig. 8f). This antibody also abolished MSC-dependent inhibition of IFN-γ production by T cells (Fig. 8h). Therefore, CCL2 production by MSCs increases T cell–MSC contact duration by enhancing T cell expression of VCAM-1. Reducing the duration of MSC–T cell contact inhibits the ability of MSCs to suppress T cell activation.

## Discussion

The results of this study provide several insights into the therapeutic mechanisms of MSCs in SLE. First, we verified that MSCs ameliorate lupus in the MRL*.Fas*^lpr^ mouse model by inhibiting cytokine production and reducing the infiltration of inflammatory cells into the kidney. Second, we showed that MSCs inhibit T cell functions *in vitro* presumably by producing several soluble immunosuppressive factors including NO, PGE_2_, TGF-β, and IDO. Third, we showed that MSC–T cell contacts enhance the inhibitory effect of MSCs on T cell function. MSCs actively recruit MRL.*Fas*^lpr^ T cells and exhibit long-term cell–cell interactions. Fourth, MSC-derived CCL2 is the critical chemokine whose production recruits T cells to MSCs. MSCs unable to secrete CCL2 cannot attract T cells, nor maintain the long-term contacts needed for T cell suppression. Fifth, CCL2 produced by MSCs upregulates the expression of VCAM-1 on MRL.*Fas*^lpr^ T cells, which enhances long-term MSC–T cell contacts.

Our data extend previous studies that have shown the beneficial effects of human and allogeneic murine MSCs on lupus-like disease progression in the MRL*.Fas*^lpr^ mouse model^21^. We showed that a single adoptive transfer of MSCs is effective as previously noted^22^,^25^,^26^. We compared three different single-dose regimens; while all three were efficient, a single adoptive transfer of 4 × 10^6^ human MSCs performed better than the transfer of fewer cells, 4 × 10^5^ or 4 × 10^4^. Single adoptive transfer of 4 × 10^6^ human MSCs decreased renal pathology and the level of anti-DNA antibodies, and improved survival. In MRL.*Fas*^lpr^ mice, T_HEF_ help produce autoantibody, T cells accumulate at sites of end-organ damage, and Treg function is defective^31^. Furthermore, boosting Treg function reduces nephritis severity in MRL.*Fas*^lpr^ mice^32^. Quantitative and qualitative defects in Tregs have been reported in lupus patients^33^,^34^. In these mice, we found increased numbers of Tregs in the spleen and kidneys of mice that received MSCs. In addition, it reduced splenic inflammatory cytokine production; reduced the percentages of splenic CD3^+ ^, CD3^+ ^B220^+ ^, and splenic plasma cells; and increased the percentage of splenic Tregs. *In vitro*, we examined the impact of MSCs on the functions of T cells isolated from MRL.*Fas*^lpr^ mice. MSCs can suppress T cell function and enhance Treg functions by producing suppressive factors^10^,^12^,^22^,^35^; consistent with these data, we showed that allogeneic MSCs produced NO, PGE_2_, TGF-β, and IDO when activated by IFN-γ *in vitro*. The production of these soluble factors likely underlies some of the immunosuppressive effects of these cells *in vivo*^36^. Interestingly, in this study, the inhibitory activity of MSCs declined when they were separated from T cells in a transwell assay, indicating the necessity of a direct cell–cell contact for optimal suppression, a result consistent with two previous reports^37^,^38^. In one of these studies, MSC–T cell contacts triggered T cell apoptosis^37^. In another study, MSC treatment with antibodies against ICAM-1 or VCAM-1 weakened inhibition of T cell proliferation, underscoring the need for cell–cell contacts^38^. Unfortunately, our *in vitro* studies as well as those of others are limited by the lack of information about the numbers of MSCs that actually engraft *in viv*o, their life span, and their localization within immune organs and tissues. Most *in vitro* studies have used a 1:10 or even a 1:1 ratio of MSCs to lymphocytes, which are unlikely to be achieved *in vivo.* Yet, potent suppressive effects of MSCs *in vivo* argue that mechanisms exist to localize lymphocytes to the MSC microenvironment.

CCL2, a potent chemokine for monocyte recruitment, is pathogenic for kidney injury in mice and patients with lupus nephritis and urine CCL2 has been considered as a biomarker candidate for lupus nephritis^25^. By using a matrix metalloproteinase, MSCs degrade CCL2 to its antagonistic variant, which suppresses plasma cell immunoglobulin production by inactivating STAT3 and inducing the transcription factor PAX^39^. MSC-derived antagonistic CCL2 variant also inhibited inflammatory Th17 cell functions in experimental autoimmune encephalomyelitis model^40^. MSCs from MRL.*Fas*^lpr^ mice and lupus patients exhibited reduced B cell–suppressive activity both *in vivo* and *in vitro* because of a decreased production of CCL2 and its antagonistic variant compared to control MSCs^25^. MSC–T cell contacts lead to Fas–FasL engagement, which increases CCL2 secretion from MSCs and triggers T cell apoptosis 2 days after co-culture. The uptake of apoptotic T cells by macrophages leads to TGF-β production by macrophages, which up-regulates Treg function^37^. Although MSCs did not induce T cell apoptosis under our 12-h imaging conditions, we cannot exclude that the driving force for T cell detachment from MSCs might be T cell apoptosis rather than an active detachment process.

MSCs ameliorate experimental autoimmune uveitis via recruiting myeloid-derived suppressor cells in a CCL2-dependent manners^41^. Overall these studies indicate that MSCs produce CCL2 for various immune cell recruitment and secrete CCL2 variant for direct inhibition of immune cells.

However, it is unclear whether CCL2 directly regulates MSC-T cell contact. To clarify it, we assessed the migration and contact dynamics of MSCs and T cells using time-lapse imaging. Although MSCs produced several chemokines including CCL2, CCL3, CCL4, CXCL10, and CXCL12, only CCL2 played a crucial role in MRL.*Fas*^lpr^ T cell recruitment, as demonstrated by the inability of CCL2-KD or *Ccl2*^−/−^ MSCs to induce T cell migration. We found that 12–14-week-old MRL.*Fas*^lpr^ T cells expressed CCR2, CCR5, and CXCR4; however, CCR2 played a dominant role in recruiting T cells to MSCs, because a CCR2 antagonist nearly completely blocked T cell migration. The CCL2–CCR2 axis also helped maintain long-term MSC–MRL.*Fas*^lpr^ T cell contacts. When MSCs and MRL.*Fas*^lpr^ T cells were placed in close physical contact, the frequency of T cell interactions with CCL2-KD MSCs and control MSCs was similar, but the average duration of the contacts was reduced. CCL2 produced by MSCs induced VCAM-1 expression in T cells, which was otherwise lacking. Blocking the interaction between VCAM-1 and VLA-4 on MSCs provided evidence that prolonged contacts augment the suppressive activity of MSCs. Thus, CCL2 produced by MSCs plays two roles: to recruit MRL.*Fas*^lpr^ T cells to the local MSC environment and to maintain contacts between MSC- T cells. Further studies will be required to address how MSCs increase VCAM-1 expression on T cells. MSCs are known to release microvesicles containing proteins, lipids, mRNA, and microRNAs; microvesicles transfer the adhesion molecule CD41 from platelets to endothelial cells^42^, implying that MSCs might be able to release VCAM-1 mRNA-containing microvesicles that might induce VCAM-1 expression on T cells. CCL2 is also known to induce VCAM-1 expression on synovial fibroblasts^43^, implying that CCL2 produced by MSCs might increase VCAM-1 expression on T cells. Our future data will help to reveal how MSCs inhibit T cells in a CCL2-dependent contact-inhibition manner.

Contact dynamics of T cells and MSCs is similar to that of T cells and dendritic cells (DCs). MSCs and DCs are sessile, but T cells actively move at a velocity of 7 μm/min; after meeting MSCs or DCs, T cells migrate slowly on the surface of MSCs or DCs^44^,^45^. Long contacts (>10 min) between DCs and T cells induce antigen-specific immunity of T cells, whereas short contacts (<2 min) occur under tolerogenic conditions^46^. In our movies, we showed that after long-lived contacts (>80 min) with MSCs, T cells weakly proliferated and had lower cytokine production. Overall, these data suggest that T cells exchange information with MSCs or DCs through contact-dependent mechanisms, but with different kinetics.

Our future studies will focus on studying the contact dynamics between MSCs and B cells. The *lpr* mutation leads to an age-dependent B cell tolerance breakdown in MRL*.Fas*^lpr^ mice^21^. B cell differentiation into autoantibody-secreting cells in these mice largely occurs at extrafollicular sites. Autoantibody production is supported by CD4^+^ T extrafollicular helper cells (T_HEF_); these cells reside outside the B cell follicle and their gene expression profile resembles that of follicular helper T (T_FH_) cells, although they express CXCR4 rather than CXCR5^47^,^48^. Yet, the mechanisms by which MSCs suppress B cell hyperactivity in MRL.*Fas*^lpr^ mice remain largely unknown. Do they target B cell activation, expansion, or differentiation? Do they restore B cell tolerance? Or do they target T_HEF_ and T_FH_ cells, or enhance Treg activity? Two recent studies focused on a direct effect of MSCs on B cells^22^,^25^. Both studies showed that a single transfer of allogenic MSCs reduced SLE in MRL.*Fas*^lpr^ mice and assessed the impact of MSCs on B cell functions *in vitro*. In co-culture experiments, MSCs suppressed MRL.*Fas*^lpr^ B cell proliferation and antibody secretion, and reduced CpG-stimulated secretion of BAFF by dendritic cells^22^. MSCs from MRL.*Fas*^lpr^ mice exhibited reduced B cell–suppressive activity both *in vivo* and *in vitro* because of a decreased CCL2 production compared to control MSCs. Interestingly, the suppressive effect of MSC-produced CCL2 depended upon its proteolytic processing by matrix metalloproteinase 1, which apparently generated inhibitory peptides^25^. CCL2 processing by MMP1 is known to generate CC chemokine receptor antagonists with anti-inflammatory activity^49^. However, it remains unknown whether sufficient numbers of MSCs reach lymphoid organs to directly target B cells, which are predominately localized in specialized niches.

In summary, we have shown the beneficial effect of human MSCs on SLE progression in MRL*.Fas*^lpr^ mice and demonstrated that allogeneic MSCs likely inhibit T cells via soluble factor- and contact-dependent pathways. Furthermore, we uncovered interesting contact dynamics between MSCs and T cells. These data reveal how MSCs inhibits T cell functions at the single-cell level and helps to understand the role of cell–cell contacts in the suppressive activity of MSCs toward activated T cells.

## Methods

### Mesenchymal stem cells

Human bone marrow (BM)–derived MSCs were obtained from Corestem Inc. (Seoul, Korea)^50^. In brief, BM was aspirated from the posterior iliac crest of healthy donors and mononuclear cells were collected by density gradient centrifugation. These cells were cultured in CSMB-A06 medium (Corestem Inc.) containing 10% fetal bovine serum (BD Biosciences, Franklin Lakes, NJ, USA), 2.5 mM l-glutamine, and penicillin/streptomycin (WelGene, Gyeonggi, Korea) in a 5% CO_2_ incubator at 37 °C for 3–5 passages. After washing out non-adherent cells, the adherent cells retained the canonical phenotype of MSCs (CD29^+^CD44^+^CD73^+^CD105^+^CD90^+^CD34^−^CD45^−^HLA-DR^−^) and were used in the experiments. All human MSC studies were approved by the Institutional Review Board of Hanyang University Hospital and carried out in accordance with their approved guidelines and all participants provided written informed consents.

Mouse MSCs were generated from the BM cells of tibiae and femurs of 6–8-week-old Balb/c or C57BL/6 mice (Orient Bio, Gyeonggi, Korea) or *Ccl*2^−/−^ (B6.129S4-*Ccl2*^*tm1Rol*^/J) mice (Jackson Laboratory, Bar Harbor, ME, USA). BM cells were cultured in α-MEM medium containing 10% fetal bovine serum, 2 mM l-glutamine, and penicillin/streptomycin in a 5% CO_2_ incubator at 37 °C. Non-adherent cells were removed on day 1 and adherent cells were cultured with medium replenishment every three days. They were used between days 17 and 20. MSCs had the surface markers Sca-1^+^CD44^+^CD73^+^CD45^−^CD11b^−^CD11c^−^Gr-1^−^MHC-II^−^ 38. The stemness of MSCs was determined by their ability to differentiate into adipocytes, chondrocytes, and osteoblasts^38^. On day 20, medium was changed. After 24 h, the levels of the soluble factors in medium were measured. NO levels were measured with Griess reagent^51^. The levels of TGF-β, CCL2, CCL5, and CXCL12 were measured by using ELISA kits (Bio-Techne, Minneapolis, MN, USA). The levels of IDO were measured by using ELISA kits purchased from BlueGene Biotech (Shanghai, China). siRNAs targeting mouse chemokines were purchased from Bioneer. MSCs were transfected with 100 nM siRNAs using Lipofectamine RNAiMAX reagent (Thermo Fisher Scientific, Waltham, MA, USA) following the manufacturer’s protocol. After 48 h, cells were used in experiments^52^. All animal studies were approved by the Chungbuk National University Animal Experimentation Ethics Committee and carried out in accordance with their approved guidelines.

### Lupus-prone MRL.*Fas* ^lpr^ mice

Female MRL*.Fas*^lpr^ (MRL.MpJ-*Tnfrsf6*^*Faslpr*^/J) mice were purchased from the Jackson Laboratory. Mice were housed in specific pathogen–free conditions at 21–24 °C and 40–60% relative humidity under a 12 h light/dark cycle. In our first experiment, MRL*.Fas*^lpr^ mice were divided into the following groups: control (vehicle, n = 5), cyclophosphamide (50 mg/kg, n = 6), and MSCs (1 × 10^6^ cells/injection, n = 6). Injections were performed intravenously 6 times every two weeks from the age of 10 weeks. In our second experiment, female MRL*.Fas*^lpr^ mice were injected intravenously with vehicle (vehicle, n = 5), cyclophosphamide (50 mg/kg, n = 5), 4 × 10^4^ MSCs/mouse (n = 5), 4 × 10^5^ MSCs/mouse (n = 5), and 4 × 10^6^ MSCs/mouse (n = 5) once at the age of 12 weeks. In our third experiment, MRL*.Fas*^lpr^ mice were injected intravenously with control (vehicle, n = 5), cyclophosphamide (50 mg/kg, n = 5), *Ccl2*^+/+^ MSCs (1 × 10^6^ cells/injection, n = 5), and *Ccl2*^*-*/*-*^ MSCs (1 × 10^6^ cells/injection, n = 5). Survival rate and body weight were examined every week. Urine and serum were collected every two weeks and stored at −70 °C until used. The levels of protein in urine and anti-dsDNA IgG and total IgG in serum were measured by using ELISA kits purchased from Sigma-Aldrich, Alpha Diagnostic International (San Antonio, TX, USA), and eBioscience (San Diego, CA, USA), respectively, according to the manufacturers’ instructions. In week 20, kidneys were isolated and fixed in 10% formalin for 3 days for immunohistochemistry.

### Time-lapse imaging

In our first imaging experiment, we examined T cell migration towards MSCs. Unstained MSCs (70 μl of 0.3 × 10^6^ cells/ml) were seeded into the left chamber and T cells (70 μl of 3 × 10^6^ cells/ml) into the right chamber of culture-insert μ-Dish^35mm^ culture dishes (ibidi GmbH, Martinsried, Germany). Time-lapse imaging was performed with a Biostation IM-Q microscope equipped with a 10x magnification objective (numeric aperture 0.5) and environmental chamber kept at 37 °C and 5% CO_2_ (Nikon Inc., Melville, NY, USA). Dishes were preincubated for 3 h in the chamber and then inserts were carefully removed. Images were acquired every 2 min for 6 h^53^. In our second imaging experiment, we examined the contact dynamics between MSCs and T cells. Cells were labeled with 5 μM carboxyfluorescein succinimidyl ester (CFSE) or 5 μM 5-(and-6)-(((4-chloromethyl)benzoyl)amino) tetramethylrhodamine (CMTMR) (Thermo Fisher Scientific, Waltham, MA, USA) in serum-free medium for 15 min at 37 °C. After staining, cells were washed twice in medium. MSCs (0.1 × 10^6^ cells/ml) and T cells (1 × 10^6^ cells/ml) were mixed and added onto 35-mm culture dishes (BD Biosciences, Franklin Lakes, NJ, USA). Dishes were preincubated for 1 h under the microscope and images were acquired in three channels (phase contrast; CFSE, green filter; and CMTMR, red filter) every 2 min for 6 h^54^. Images were analyzed by using Imaris software version 7.2 (Bitplane, Zurich, Switzerland). Cells were automatically tracked by using spot analysis with the autoregressive motion algorithm, broken tracks were manually reconnected, and background spots were manually removed^55^. Instantaneous velocity was calculated automatically by Imaris software. The number of contacts that lasted for >6 min was determined.

### T cell functions

T cells were purified from spleen cells of MRL*.Fas*^lpr^ mice by a negative depletion method using biotinylated-antibodies specific for B220, GR-1, and CD11c (BD Biosciences) and Dynabeads M-280 Streptavidin (Thermo Fisher Scientific)^56^. Purity was typically >90%. To measure cell proliferation, purified T cells (1 × 10^5^ cells/well) and MSCs (0.01–0.1 × 10^5^ cells/well) were mixed in 96-well plates. Sometimes, MSCs were cultured in the upper well and T cells in the lower well of transwell plates (BD Biosciences) to avoid cell–cell contact. Concanavalin A (Con A, 1 μg/ml) was used to specifically activate T cells. Cells were pulsed with ^3^H-thymidine (113 Ci/nmol; NEN, Boston, MA, USA) at a concentration of 1 μCi/well for the last 18 h and were harvested on day 3 using an automated cell harvester (Inotech, Dottikon, Switzerland). The amount of ^3^H-thymidine incorporated into cells was measured using a Wallac Microbeta scintillation counter (Wallac, Turku, Finland)^56^. The levels of T cell-derived cytokines (IFN-γ and IL-2) were determined by using an ELISA kits (Biotechne). For chemotaxis assays, T cells were added in a volume of 100 μl to the upper wells of transwell plates with a 5-μm insert (Corning). Various concentrations of chemokines or MSCs were added to the lower wells in 600 μl of complete RPMI 1640 medium. The number of T cells migrated to the lower well over 1.5 h was counted using a flow cytometer. Sometimes, T cells were pre-incubated with the CCR2 antagonist RS102895 (3–30 μg/ml) for 1 h.

### Cell conjugation assay

T cells (1 × 10^6^ cells/ml) were labeled with 0.5 μM 5-chloromethylfluorescein diacetate (CMFDA) (Life Technologies) and MSCs with 5 μM 5-(and-6)-(((4-chloromethyl)benzoyl)amino) tetramethylrhodamine (Thermo Fisher Scientific) in serum-free medium for 10 min at 37 °C. After staining, cells were washed twice in culture medium having 10% FBS. MSCs (1 × 10^5^) and T cells (1 × 10^6^) were mixed in a 12 × 75-mm polystyrene tube (BD Biosciences) and centrifuged at 1000 rpm for 1 min, and pellets were incubated at 37 °C for 2 h. Cell mixtures were then gently suspended and analyzed by flow cytometry. The conjugation ratio was calculated as the portion of CMFDA/CMTPX double-positive events.

### RT-PCR, western blotting, and flow cytometry

These experiments were performed as previously described^52^. Cells were analyzed using a FACSCalibur flow cytometer and data were processed using Cell Quest Pro software (BD Biosciences). Forward and side scatter parameters were used to gate live cells; 10,000 events were analyzed^52^.

### Statistical analysis

Data represent the mean ± SEM of at least three independent *in vitro* experiments performed in triplicates or at least five mice. To determine statistical significance, *p* values were calculated using one-way ANOVA (GraphPad Software, San Diego, CA, USA). Some data represent the mean ± SEM of T cells (Figs 6c,e,f and 8f,g) and *p* values were calculated using Mann-Whitney test (GraphPad Software, San Diego, CA, USA).

## Additional Information

**How to cite this article**: Lee, H. K. *et al*. CCL2 deficient mesenchymal stem cells fail to establish long-lasting contact with T cells and no longer ameliorate lupus symptoms. *Sci. Rep.* **7**, 41258; doi: 10.1038/srep41258 (2017).

**Publisher's note:** Springer Nature remains neutral with regard to jurisdictional claims in published maps and institutional affiliations.

## Supplementary Material

Supplementary Movie S1

Supplementary Movie S2

Supplementary Movie S3

Supplementary Movie S4

Supplementary Movie S5

Supplementary Movie S6

Supplementary Movie S7

Supplementary Movie S8

Supplementary Movie S9

Supplementary Movie S10

Supplementary Movie S11

Supplementary Movie S12

Supplementary Figures
