# Supplementary material for: CCL2 deficient mesenchymal stem cells fail to establish long-lasting contact with T cells and no longer ameliorate lupus symptoms
Source: Sci Rep. 2017 Jan 24;7:41258. doi: 10.1038/srep41258 (PMC5259742; doi:10.1038/srep41258)
Supplement: Supplementary Figures [file srep41258-s13.pdf]

Supplementary Figures

CCL2 deficient mesenchymal stem cells fail to establish long-lasting contact with T cells and no longer ameliorate lupus symptoms

Hong Kyung Lee[1,†], Hyung Sook Kim[1,†], Ji Sung Kim[1], Yong Guk Kim[1], Ki Hwan Park[1], Jae Hee Lee[1], Ki Hoon Kim[1], In Young Chang[2], Sang-Cheol Bae[3], Youngsoo Kim[1], Jin Tae Hong[1], John H. Kehrl[4], and Sang-Bae Han[1,*]

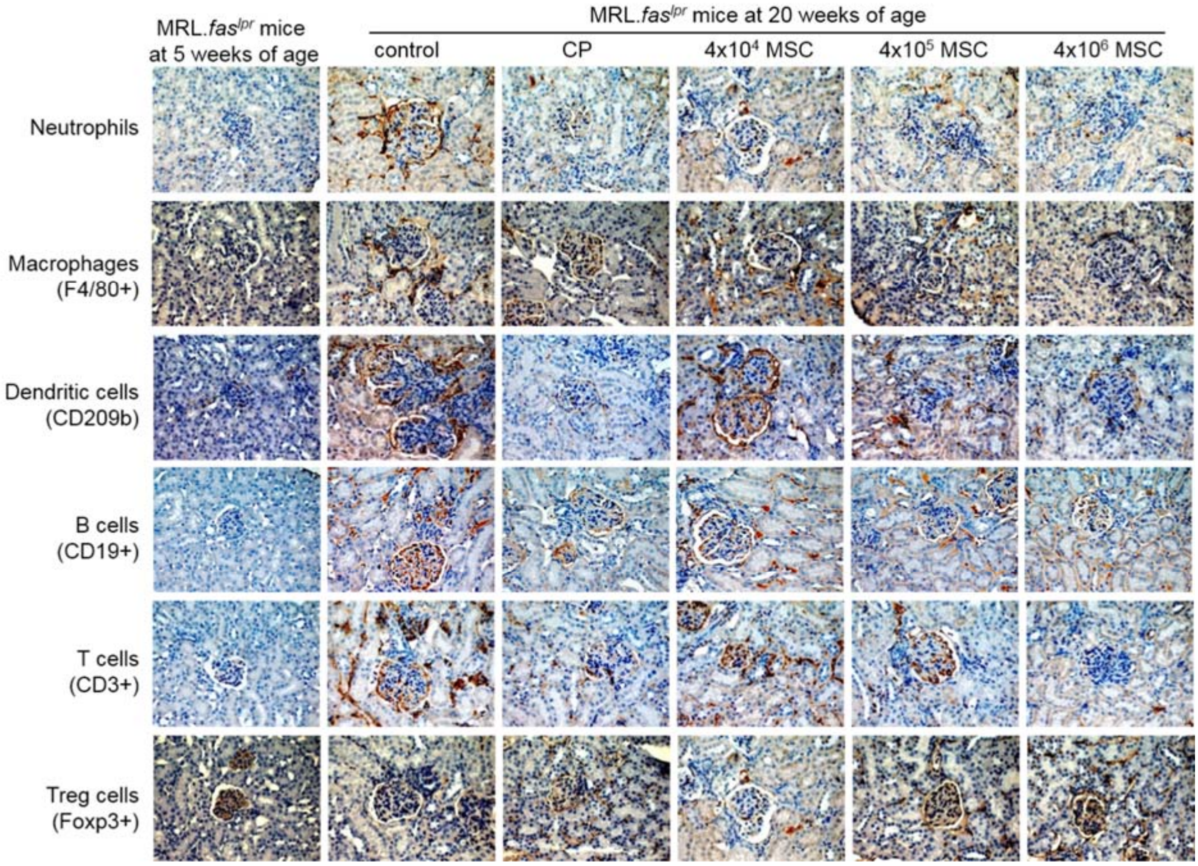

Figure S1. Immunohistochemical analysis of kidneys of MRL.*fas*^{lpr} mice. Kidneys were isolated from mice used in Figure 2 and used in immunohistochemical analysis.

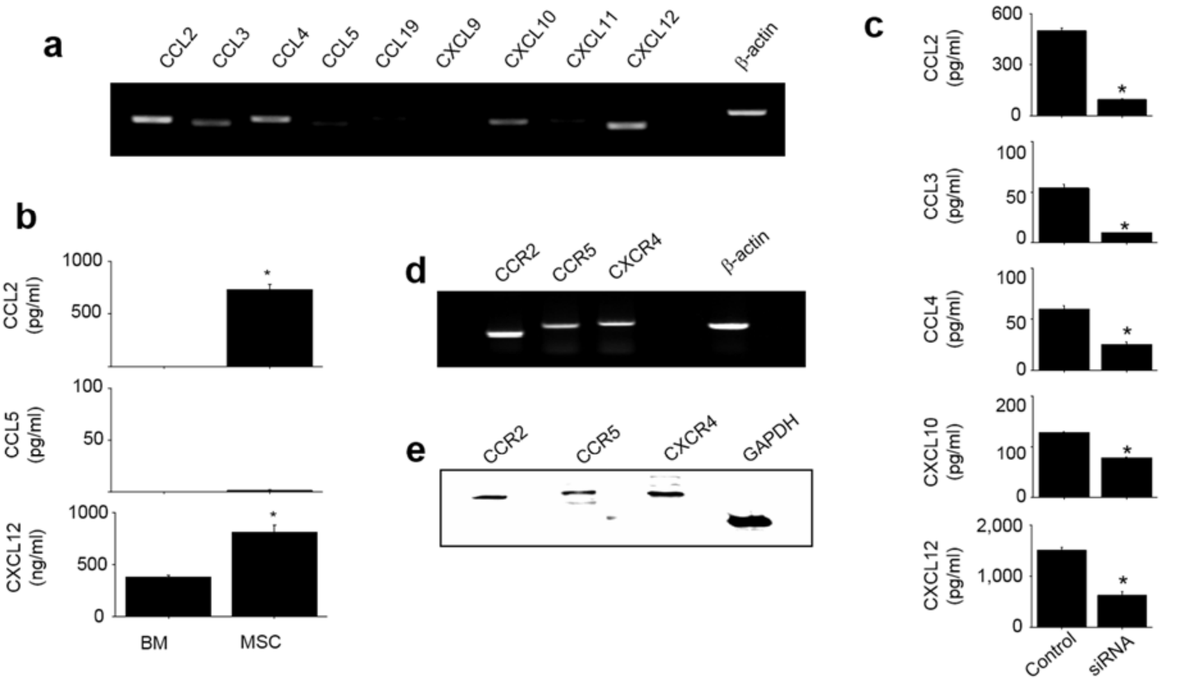

Figure S2. Chemokine expression in MSCs and chemokine receptor expression in T cells. (a, b) Total RNA was isolated from MSCs on day 20 and chemokine gene expression was examined by RT-PCR (n = 3) (**a**). The levels of chemokines accumulated in culture medium over 24 h (days 20–21) were determined by ELISA (n = 3) (**b**). (**c)** MSCs were transfected with siRNAs to decrease the expression of several chemokines, and chemokine levels were determined by ELISA after 48 h. * $p < 0.01$. (**d)** MRL.*Fas*^{lpr} T cells were activated with concanavalin A (Con A, 1 μg/ml) for 72 h (n = 3). The expression of chemokine receptors was examined by RT-PCR (RT) and western blotting (WB).

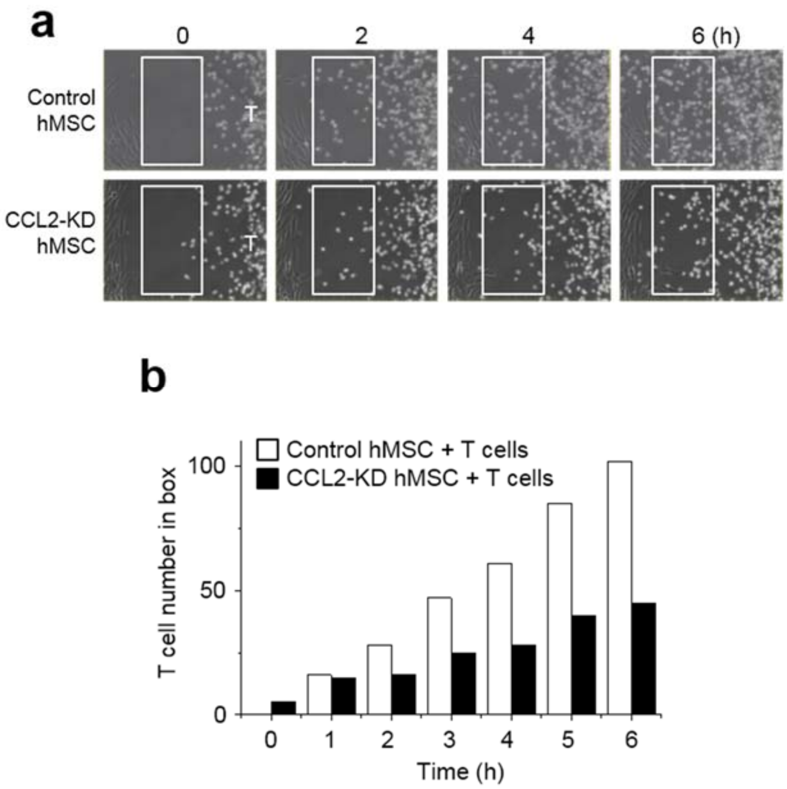

Figure S3. T cell migration towards human CCL2-KD MSCs.
Human MSCs (hMSCs) were transfected with negative (control) or CCL2 siRNA (CCL2-KD). T cells were purified from spleens of MRL.*Fas*^{lpr} mice. For time-lapse imaging, MSCs (70 µl of 0.3 × 10^6 cells/ml) were seeded into the left chamber and T cells (70 µl of 3 × 10^6 cells/ml) into the right chamber of a culture insert µ-Dish^{35mm} culture dishes. Images were acquired every 2 min for 6 h (n = 2). (**a**) Representative snapshots. (**b**) The number of T cells passing through the white boxes.

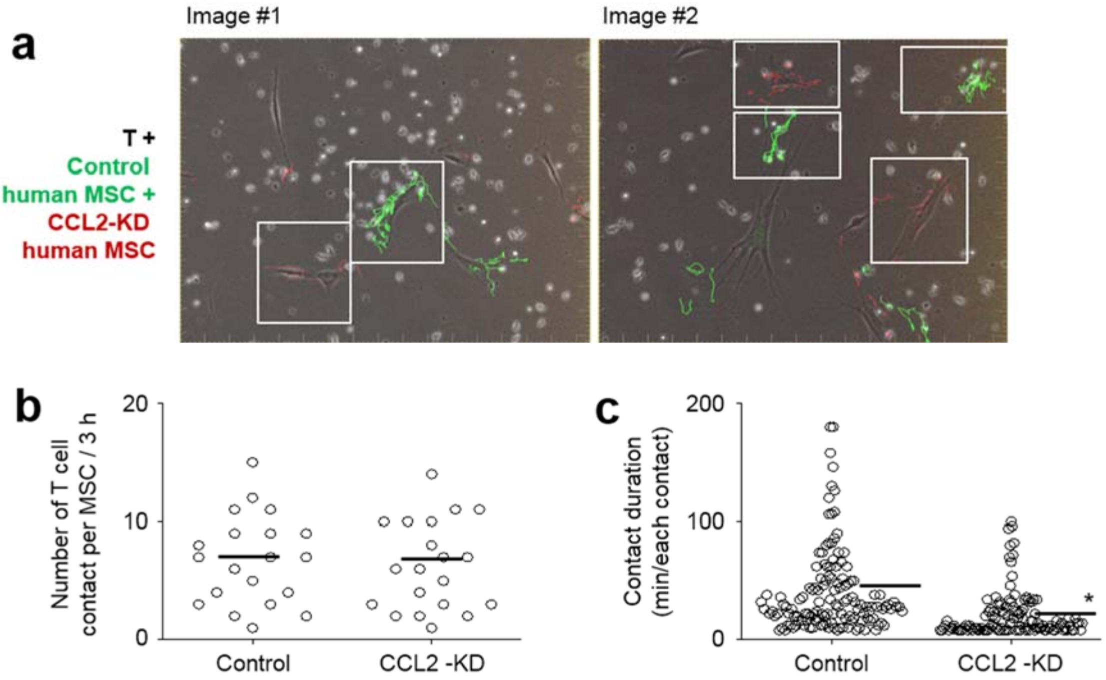

Figure S4. Contact dynamics between T cells and human MSCs.
Human control MSCs were labeled with CFSE (green) and human CCL2-KD MSCs were labeled with CMTMR (red). Control (0.05×10^5 cells/well) and CCL2-KD MSCs (0.05×10^5 cells/well) and T cells (1×10^5 cells/well) were mixed and added onto 35-mm culture dishes. Dishes were preincubated for 4 h under the microscope and images were acquired in three channels (phase contrast; CFSE, green filter; and CMTMR, red filter) every 2 min for 3 h (six movies from three independent experiments per group). Images were analyzed by using Imaris software. (**a**) Representative images. (**b**) The number of T cell contacts per MSC (n = 20 and 20 from the left). (**c**) Duration of contacts between T cells and MSCs (n = 135 and 125 from the left). Bars represent the mean of the data. $^{*}p < 0.01$ versus control.
